# Identifying chromatin features that regulate gene expression distribution

**DOI:** 10.1038/s41598-020-77638-2

**Published:** 2020-11-25

**Authors:** Thanutra Zhang, Robert Foreman, Roy Wollman

**Affiliations:** 1 Institute for Quantitative and Computational Biosciences, UCLA, Los Angeles, CA USA; 2 Departments of Integrative Biology and Physiology and Chemistry and Biochemistry, UCLA, Los Angeles, CA USA

**Keywords:** Cellular noise, Chromatin

## Abstract

Gene expression variability, differences in the number of mRNA per cell across a population of cells, is ubiquitous across diverse organisms with broad impacts on cellular phenotypes. The role of chromatin in regulating average gene expression has been extensively studied. However, what aspects of the chromatin contribute to gene expression variability is still underexplored. Here we addressed this problem by leveraging chromatin diversity and using a systematic investigation of randomly integrated expression reporters to identify what aspects of chromatin microenvironment contribute to gene expression variability. Using DNA barcoding and split-pool decoding, we created a large library of isogenic reporter clones and identified reporter integration sites in a massive and parallel manner. By mapping our measurements of reporter expression at different genomic loci with multiple epigenetic profiles including the enrichment of transcription factors and the distance to different chromatin states, we identified new factors that impact the regulation of gene expression distributions.

## Introduction

Gene expression variability is prevalent across multiple organisms ranging from bacteria to mammalian cells^1,2^. Expression variability across a population of genetically identical cells drive phenotypic diversification which is important for many biological processes including multicellular development, cell differentiation and lineage decisions, viral decision making as well as bacteria and cancer cell survival during environmental stress^3–9^. Factors contributing to cell-to-cell variability are generally classified into intrinsic and extrinsic sources^10,11^. Lately, our understanding of the molecular regulatory mechanisms underlying the stochasticity in gene expression is increasing. Several lines of evidence support significant roles in expression variability for the presence of TATA-box^12–15^, nucleosome occupancy and chromatin remodeling^11,16–18^, transcriptional pausing^19,20^, chromatin epigenetics^21–25^ and concentration of transcription factors^26,27^. However, given the numerous layers of chromatin regulation of gene expression, the lack of systematic interrogating of the impact chromatin microenvironment on gene expression variability limits our understanding of the underlying molecular factors.

The influence of local chromatin environment on gene regulation or position-effect variegation has been extensively studied since the classical work in *Drosophila* eyes in the 1930s^28–32^. Although previous studies mostly examined such effects on averaged mRNA or protein productions, positional effects on the heterogeneity of expression are understudied. The pioneering work that addresses this question in a systematic manner was done by Chen and Zhang in *Saccharomyces cerevisiae* and suggests the association between expression variability and three histone modifications, H3K4me1, H3K4me3 and H3K79me3^33^. Even though yeast and mammals share several features of transcription regulatory mechanisms, there are substantial differences in the complexity of genomes between these two^34–36^. The yeast genome, which consists of ~ 12 Mb, is extremely compact while human genome is much bigger, or about 275 times the size of the genome of yeast, and contains large amounts of noncoding DNA^37–39^. Considering additional differences in large-scale chromatin dynamics such as long-range chromatin interactions and higher-order chromatin structure, there is a need to further investigate this question in higher eukaryotes. For example, CTCF, a transcription factor conserved from fly to human but absent from yeast^40,41^, is a critical regulator who creates boundaries between topologically associating domains (TAD) and regulates gene expression variability through mediating enhancer-promoter interaction^42–44^. Few studies demonstrated that the “position effect” extends to gene expression variability in addition to its effect on average expression. Recently, Dar et al. and Dey et al. showed that an identical expression reporter cassette integrated into different genomic loci in human cell line will have different expression variability. However, due to technical challenges in identification of genomic position of these integration reporters at scale, the integration sites of these reporters were not detected limiting the ability to identify correlation between chromatin microenvironments and gene expression variability^18,45,46^.

The investigation of position effect was done in two distinct approaches with complementary benefits. Both approaches are based on the integration of identical expression reporter cassette to multiple positions in the genome. The first approach generates multiple cell lines, one for each integration reporter, either in a targeted fashion or through the random insertion of reporters from transposon- or virus-based transfection, followed by reporter mapping^33,47–51^. Once cell lines are created they provide rich data on gene expression distribution and how it correlates with different chromatin features. However, the creation of cell lines is very laborious and scale poorly. A recent study that used this approach was limited to 6 cell lines^46^ and due to this small sample size were unable to gain insights into the impact of different chromatin features. The second approach takes advantage of recent advances in DNA synthesis and next-generation sequencing technologies (NGS) to offer novel and rapid ways to interrogate chromosomal position effects on the scale of thousands of positions^52,53^. At the core of the second approach is the use of pooled assays enabled through the addition of a unique DNA barcode to the expression reporter. NGS is then used to measure gene expression averages and identify integration sites. The ability to investigate thousands of positions provided the statistical power to identify the molecular underpinning of positional effects. However, the reliance on NGS and pooled assays throughout the study limit the output of this approach as they only allow the measurement of populational mRNA average from each location. Therefore, they do not provide information about expression variability, an important feature of gene regulation.

Here we develop a new approach for the systematic investigation of position effects on gene expression variability that integrates the benefits of the two existing approaches described above. We developed a high-throughput method to build and characterize a library of isogenic clones as a platform to study the positional effects on gene expression variability at a large number of identified genomic loci. Significant levels of positional effects on gene expression mean and variance were observed across human K562 cells. By leveraging publicly available data of the K562 epigenome and mapped to our reporter measurement, we identified and key chromatin features factors that correlate with gene expression mean and variance. Our findings provide a deeper understanding of the mechanisms underlying the stochasticity in gene expression and provide the foundation for future work on the specific features identified here.

## Result

### Scalable generation and identification of isoclonal reporter clones

**Figure 1:**
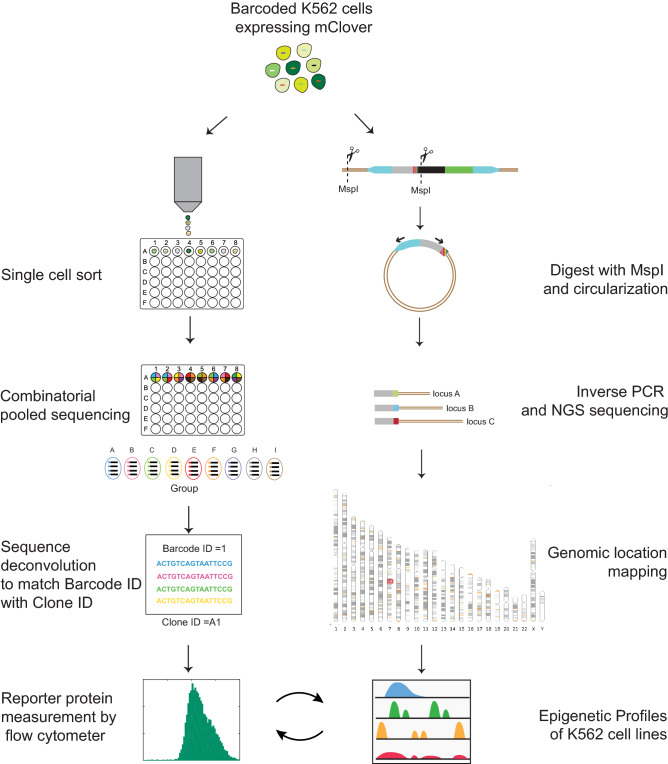
Protocol for scalable and parallel measurement of expression distribution and genomic position of reporter genes. (**A**) Two high-throughput methods were connected by incorporating random 16-bp DNA barcodes in the reporter cassette containing identical CMV promoter and a gene coding for fluorescent protein Clover. Synthetic reporter was introduced into K562 cells through low m.o.i. lentiviral transduction. (**B**) Pooled identification of the genomic position of reporter genes based on the unique DNA barcode using TRIP method. Extracted genomic DNA of mixed founder cells was digested with MspI and followed by inverse PCR and deep sequencing. (**C**) The creation of isogenic cell line, the identification of the DNA barcode sequence unique to each cell line using combinatorial pooled sequencing and the measurement of reporter expression distribution by high-throughput flow cytometry. The coupling of these two measurements allows the generation of large scale data on the effect of chromatin environment on gene expression variability.

To obtain genomic scale data on the effect of chromatin environment on gene expression variability, we developed a new approach to facilitate the creation and identification of isogenic reporter clones in a massively parallel and highly scalable manner. The overview of our method is visualized in Fig. 1. The principle of this method involved tagging individual genomic location with the reporter cassette that contains a unique 16-nucleotide DNA barcode and fluorescent reporter mClover driven by a CMV promoter. These barcodes serve as molecular identifiers for mapping the genomic location of the reporter in individual isogenic clones. The barcoded reporters were introduced into K562 cells through lentiviral transduction with a low multiplicity of infection (MOI) to ensure a single integration of reporter per cell. The founder cells were sorted by fluorescence-activated cell sorter (FACS) and expanded for two weeks and then split into two groups. The first group was used to establish isogenic clones and identify their corresponding barcodes through combinatorial pooled sequencing^54–56^. In the pooled sequencing group, each clonal identity is transformed into a unique pooling pattern. NGS is then used to map all the observed pooling patterns to the DNA barcode thereby providing key information of what DNA barcode exists in each individual cell line without the need for laborious isolation of DNA from each cell line. This form of combinatorial decoding scales as the logarithm of the number of cell lines providing a scalable way to create a large number of cell lines with known insertion barcodes. Once clonal lines were established, the measurements of reporter expression were performed by high-throughput flow cytometry, providing full information on gene expression distribution for each barcoded reporter. The other half of the founder cells were used for parallel mapping of reporter integration sites by applying Thousand of Reporters Integrated in Parallel (TRIP) method^52^ which is based on inverse PCR^57^ coupled with next-generation sequencing. After matching the detected location of barcodes with the database of K562 epigenetic profiles, information about local chromatin landscape surrounding each barcode was obtained. We confirmed the accuracy of our method through analysis of randomly picked 5 clones of barcoded cells, extracted genomic DNA, performed targeted PCR and Sanger sequencing. As expected, all revealed barcodes from Sanger sequencing matched with those deconvoluted from combinatorial pooled sequencing. We coupled these two measurements to generate large scale data for investigating the effect of chromatin environment on gene expression variability and interrogating the underlying molecular mechanisms.

### Positional effects on multiple features of gene expression

In order to achieve sufficient statistical power to predict the molecular contributors underlying each feature of gene expression, we generated a library of reporter cells on the scale of hundreds of isogenic clones. About thirty percent of cell lines that we established lost reporter expression after two weeks of clonal expansion suggesting their insertions into heterochromatic environments^58^. Since we focus on gene expression variability, these non-expressing clones were excluded from future analysis yielding a total of 90 isogenic clones with confidently mapped reporters.

**Figure 2:**
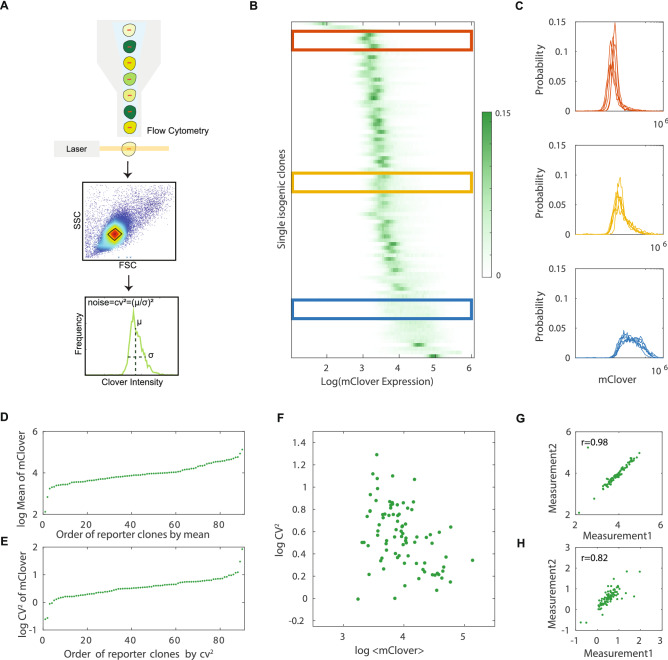
Gene expression distribution of a library of isogenic cell lines expressing CMV driven mClover fluorescent reporter shows dramatic gene expression variability. (**A**) Reporter expression at the single-cell level was measured by high-throughput flow cytometry for three replicates. A conservative gate controlling cell size, volume and cycle were applied to 50,000 live cells collected from each isogenic clone to minimize extrinsic noises. Gene expression variation can be quantified by CV^2^ which is a measure of noise independent of gene expression levels. (**B**) Stacked probability density function of log10 expression of mClover in 90 cell lines. Each row in the heatmap represents a single histogram with the probability density function color coded. (**C**) Examples of the histogram of 30 cell lines from the top (red), middle (yellow), and bottom (green) of the stack in (**B**). (**D**, **E**) The mean (**D**) and noise (**E**) of Clover levels in 90 isogenic clones. Each point represents averaged fluorescence intensity (**D**) or the intrinsic noise CV^2^ (**E**) of Clover of a single reporter clone representing the data from one specific genome location. (**F**) Scatter plot of the measured mean and noise across positions. For clarity extreme points shown in (**D**) and (**E**) are omitted. Axes are on a logarithmic scale. (**G**–**H**) The mean (**G**) and noise (**H**) of Clover expression of each isogenic clone is plotted for two measurement replicates (Spearman's rank-order correlation coefficient is 0.98 for expression mean (**G**) and 0.82 for expression noise (**H**); both axes are in logarithmic scale). Each measurement was performed independently on different experimental setup and dates.

Established isogenic clones were next profiled for reporter expression at single-cell resolution by high-throughput flow cytometry. Specifically, three replicate measurements were performed on three different dates for each clone. We monitored and controlled batch effects between measurement by co-culturing control cells expressing both Clover and IRFP protein with experimental clones. Forward scatter (FSC), side scatter (SSC), Clover signal and IRFP signal of 50,000 lived cells were collected. To minimize extrinsic noise from differences in cell size, we applied a conservative cell selection criteria that was validated previously in several studies to attenuate extrinsic noise^59–62^ using a small gating on the FSC versus SSC for a subset of live cells. Clover intensity of experimental clones was isolated from internal control cells by IRFP gating and calculated for expression average and noise (Fig. 2A). We chose to quantify protein expression noise by the squared coefficient of variation (CV^2^), which is defined as the ratio of variance over the mean squared, as it was widely used in several experimental systems and studies of gene expression noise^59,63–66^.

A direct comparison of reporter expression across 90 clones shows dramatic variability in the distribution of Clover protein (Fig. 2B and 2C). We observed ~ 1000 fold difference in mean reporter protein level between the dimmest and the brightest clone (Fig. 2D). Reporter expression noise from CMV promoter is altered more than 300 times by their positions (Fig. 2E). The CV^2^ of the most variable clone is ~ 85 while the most quiet one is only ~ 0.25. As expected, CV^2^ decreases with an increase in expression mean (Fig. 2D). However, substantial variability in CV^2^ cannot be explained solely based on expression mean suggesting that the stochasticity in gene expression is also position dependent (Fig. 2F). Expression data are highly correlated between independent measurements (Fig. 2G and 2H). Moreover, expression distributions of internal control cells are highly consistent across 90 clones discounting the possibility that observed effects arise from technical noises due to culture conditions. Overall, our data show a comparable level of the positional variation in expression average and variability when compared with other studies using higher eukaryotes as a model system^45,52^ supporting the significant contribution of position effects on multiple aspects of gene expression distributions.

### Integrative analysis of transcription factors contribution to gene expression mean and CV^2^

**Figure 3:**
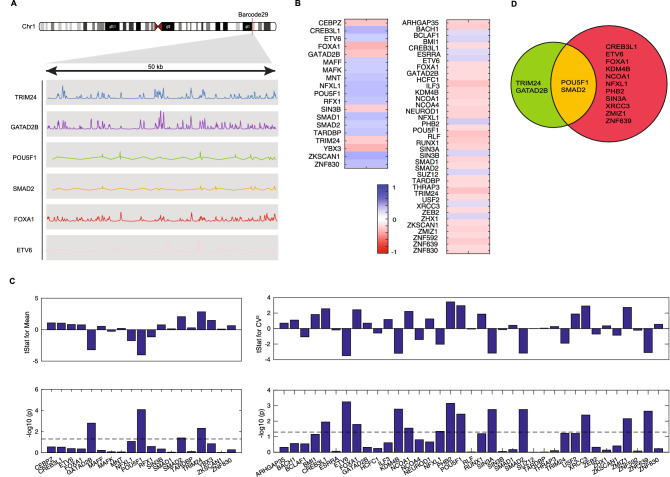
Identification of key transcription factors associated with reporter expression levels and noises using multivariate linear regression analysis. (**A**) View of the barcode 29 locus on chromosome 1. Genome coordinate of each barcode was obtained from conducting TRIP experiment. Six examples of normalized ChIP-seq signal profile of transcription factor surrounding the integration site of the barcode 29 were visualized for a domain of 50 kb. (**B**) The heatmap showing the correlation between the enrichment of transcription factors and reporter expression mean and noise. Transcription factors with Spearman's rank-order correlation coefficient of more than 0.2 were selected from over 200 tested transcription factors. (**C**, **D**) To understand the relationship between the enrichment of TF and the expression levels (**C**) and noise (**D**) of reporter in an integrative way, we selected transcription factors showing a significant correlation in (**B**) to fit multivariate linear regression model. Features with a significant level above the threshold or dashed line (p < 0.05) contributed significantly to the model. (**E**) A Venn diagram listing transcription factors contributing to reporter expression mean (green) and noise (red).

To better understand the molecular mechanisms underlying the position effects on expression level and noise, we integrated publicly available high-quality ChIP-seq data from ENCODE^67,68^ with our measurement data. Specifically, a window of 50 kilobases surrounding the position of each barcode was used to calculate the enrichment of transcription factor (TF) (Fig. 3A) surrounding each reporter. The Spearman rank correlation coefficient between TF enrichment and expression mean and CV^2^ measured by our assay was calculated. From about two hundred tested transcription factors, only a small number of them showed a moderately positive or negative correlation with expression mean and noise (Fig. 3B).

To identify a subset of TFs that play a role in gene expression we used multivariate linear regression analysis with a stepwise procedure to determine the terms in the model to integratively identify the most likely candidate transcription factors linked to our reporter activities. Model selection approach was required due to the large number of TFs with high-quality ChIP-seq data publicly available. This approach identified a set of transcription factors that statistically explain the reporter expression average and CV^2^, with an estimate of their relative contribution to the predictive power of the model (Fig. 3C, 3D). Interestingly, we found transcription factors controlling reporter expression average do not always mutually regulate expression CV^2^ (Fig. 3D) and suggest specific factors that orthogonally control two features of gene regulation.

Many of the identified contributing transcription factors have functions involved in chromatin remodeling and transcriptional activation or repression. For example, GATAD2B is one of the factors contributing to the level of reporter expression average. Higher enrichment of this TF is correlated with lower mean of reporter expression. GATAD2B, encoded from the human GATA zinc finger domain-containing 2B is beta‐subunit of the transcription repressor complex MeCP1‐Mi2/nucleosome remodeling and deacetylase complex that involved in chromatin modification and transcription activity^69–71^. TRIM24, on the other hand, has an opposite effect on reporter expression average. Higher enrichment of this TF is associated with increased reporter expression. Previous studies have reported TRIM24 as a transcriptional activator in various signaling pathways^72–74^.

Moreover, our results also indicate a possible role of pioneer transcription factor in gene expression noise. For instance, FOXA1, forkhead box protein A, significantly correlates with the variability of reporter expression. This transcription factor is postulated to have unique properties that allow them to interact with closed nucleosome arrays, initiate epigenetic switch and thereby open condensed chromatin structures^75–78^. Additionally, we found POU5F1, also known as OCT4, is correlated with high CV^2^. POU5F1 possess DNA binding domain that differs from that of FoxA, but also preferentially target silent sites enriched for nucleosomes. As a result, its pioneer activity can initiate cell-fate changes^79,80^. Accordingly, a previous study found genes with super-enhancers that are densely occupied by POU5F1 have unusually high cell-to-cell expression variation^81^.

### Distance to chromatin states influences expression mean and CV^2^

K562 cell line is a well-established model for studies of chromatin regulation and has the largest number of publicly available datasets generated mainly by the ENCODE project^82^. Although the individual data track of epigenetic marks and the regulatory element is informative, the systematic annotations derived from their interrelations contain higher-level information and provide deeper insight into the functional elements of the genome. Therefore, we examined the influence of chromatin states on reporter expression levels. Chromatin states of K562 used in our analysis were learned by computationally integrating ENCODE ChIP-seq, DNase-seq, and FAIRE-seq data using a Hidden Markov Model (HMM)^83^. Whole-genome of K562 were segmented into twenty-five states according to the combination of multiple epigenetic marks and these states were then classified into ten predicted functional elements including active promoter (Tss,TssF), promoter flanking (PromF), inactive promoter (PromP), candidate strong enhancer (Enh, EnhF), candidate strong enhancer or DNase (EnhWF, EnhW, DNaseD, DNaseU, FaireW), distal CTCF or candidate insulator (Ctcf, CtcfO), transcription associated (Gen5′, Elon, ElonW, Gen3′, Pol2, H4K20), low activity proximal to active states (Low), polycomb repressed (ReprD, Repr, ReprW) and heterochromatin (Quies, Art)^84^.

**Figure 4:**
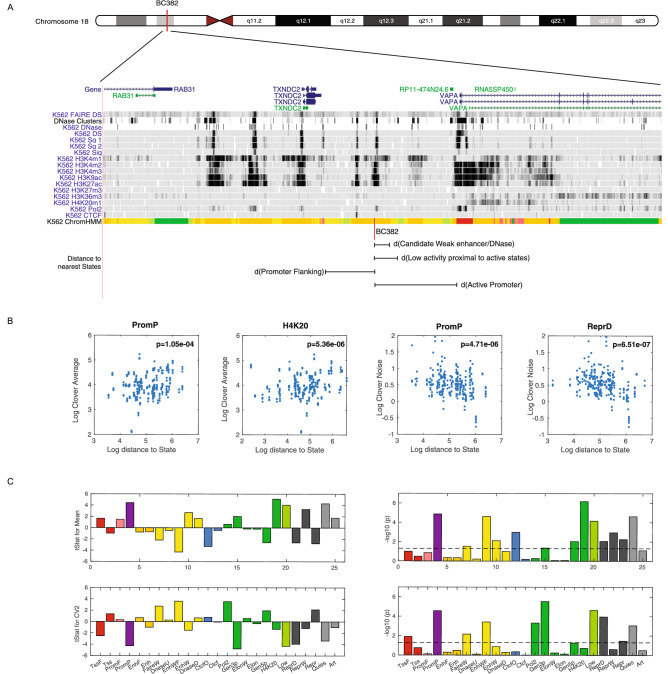
Distance to specific chromatin states influences expression mean and variance (**A**) Nearest distance from the barcode location to each chromatin state segmented by ChromHMM method was calculated. (**B**) The correlation plot between distance to specific chromatin state and reporter expression mean or noise. (**C**) A multivariate linear regression model was used to determine significant chromatin states influencing reporter expression mean and noise. Panels show the student t statistics and p-values of each coefficient in the model for expression mean (top) and noise (bottom). Chromatin states are color-coded.

We calculated the nearest distance between reporter location to each chromatin state (Fig. 4A) and applied multivariate linear regression to collectively investigate the contribution of the distance of chromatin state to reporter expression (Fig. 4C). We found reporter expression average was significantly associated with the distance to the following states: H4K20, PromP, Quies, EnhWF, Low, CtcfO, ReprW, Repr, EnhW, ReprD, Gen5p, FaireW, and Gen3p respectively. Only a subset of these chromatin states displayed their connection with reporter expression CV^2^. The significant contributors to expression CV^2^ include chromatin state Gen3p, Low, PromP, ReprD, EnhWF, Pol2, Quies, FaireW, TssF, and Repr successively. We found the distance to chromatin state H4K20 and CtcfO is highly associated with gene expression mean but not the noise. Reporter genes located closer to CtcfO trend to have higher averaged gene expression. CtcfO is highly enriched in CTCF, PolymeraseII, H3K4me1 and a marker of opened chromatin (DNase). Oppositely, closer distance to H4K20 is likely to decrease expression mean. This relationship is in agreement with several studies previously found the role of H4K20 methylation in transcriptional repression and gene silencing^85–87^.

Interestingly, two chromatin states that are highly associated with expression variability detected by our assays (Fig. 4B) are linked with bivalent chromatin structure. PromP is ‘poised promoter’ and associated with both the active H3K4me3 mark and the polycomb-repressed H3K27me3 modification. ChromHMM state ReprD has a relatively high frequency of H3K27me3 and DNase sensitivity. Recently, two research groups have reported conflicting chromatin states as one of the determinants of high noise in gene expression^20,81^. Therefore, our results are consistent with previous studies. Moreover, we also found highly significant contribution of the distance to chromatin state Pol2 and Gen3p to gene expression CV^2^ and not expression mean. Although these two states possess similar profiles of high Polymerase II enrichment and relatively open chromatin structure, the contrasting effects on expression variability were observed. Gen3p state is associated with high expression noise and Pol2 state is vice versa. The key differences of epigenetic marks between these two states are H3K36me3 and H4K20me1 suggesting their roles in enhancing noise in gene expression.

## Discussion

Here, we developed a new method to investigate the underlying factors controlling gene expression mean and variability in the human genome (Fig. 1). We showed that the insertion site affects multiple aspects of gene expression distributions (Fig. 2). Mechanistic insights related to the factors underlying expression mean and variance noise were gleaned by leveraging multiple epigenetic profiles with our measurement data (Figs. 3 and 4). Overall, our results provide new insights into chromatin factors that contribute to regulation of gene expression and highlights the importance of chromosomal context in gene regulation.

Our results illustrate the power of combining two high-throughput sequencing-based tools. TRIP has high capacity of revealing several thousands of barcodes in a single run of deep sequencing while the use of combinatorial pooled sequencing allows the identification of DNA insertion in cell line in a scalable manner that eliminate the process of individual genomic extraction and PCR amplification per clone. Moreover, our approach is highly scalable. Identification of thousands of clones can be achieved by only tens of genomic extraction and PCR through encoding clone identity in a format of pooling pattern. For instance, 24-choose-4 or 10,626 cell lines can be pooled into 24 flasks in a specific combinatorial pattern such that each cell line is combined into a unique subset of exactly four flasks out of the 24 flasks. Therefore, the only limit of scale is pipetting time to pool cell lines and measure gene expression. This bottleneck can be overcome through the use of advanced robotic pipettor^88^.

Nonetheless, it is important to note that the work presented here does have key limitations. While the framework presented could be scalable in principle, the work presented here is only based on 90 clones. It is likely that many chromatin features were missed in the present analysis. It is furthermore possible that unforeseen technical issues as well as conceptual bottleneck could limit its ability to scale. Additional limitations stems from our use of k562 cells. The choice of this experimental model system has advantages: (1) because it is one of the major ENCODE cell lines and a substantial amount of epigenomic data was already collected for this cell. (2) Due to it’s transformed nature, it is amenable to experimental manipulation. However, we anticipate that additional work will be needed to apply our approach for other experimental systems that do not share these benefits. System with limited availability of epigenomic data will be challenging as it will be hard to interpret the position specific expression noise patterns. Systems that are hard to manipulate to create the cell line needed will also be very difficult to implement for obvious technical reasons. Finally, given that K562 is a leukemia cell line, future work will be needed to evaluate how generalizable our results are to non transformed cells.

The discovery of DNA methylation and histone post-translational modifications has led to extensive studies on the impact of epigenetic modifications on gene regulation. Although earlier studies generally considered each modification as a simple code, further exploration revealed complex patterns of their combinations. Recently, the concept of chromatin state, defined by the combinatorial presence and absence of multiple marks, has been introduced into epigenetic field to facilitate the functional interpretations of distinctive genome characteristics. Intuitively, local chromatin environment can be viewed as a unique combination of these basic building blocks. Yet how diversifying the organization of these blocks affect gene expression remains elusive. By directly quantifying the relationship between the distance to chromatin states and gene regulation, we are able to provide functional annotation to chromatin state in a way that is not influenced by the underlying DNA sequence. Our analysis recapitulates the previously reported roles of bivalent chromatin on gene expression variability. Additionally, we found that the distance to chromatin states CtcfO and H4K20 independently control reporter expression mean, while the distance to chromatin states Pol2 and Gen3P solely control reporter expression noise. Further investigation should focus on the mechanistic roles of H3K36me3 and H4K20me1 in gene expression variability. Since previous studies have observed that these two marks could regulate RNAPII-catalyzed transcription elongation by recruiting specific elongation inhibitors and enabling dynamic changes in chromatin compaction^89–92^, it is possible that modifying these histone marks could control gene expression noise through adjusting transcriptional elongation rate.

Chromatin regulation of gene expression is a highly complex process that is tightly coordinated with numerous transcription factors. It is now widely accepted that transcription factors modulate gene expression through multiple modes of mechanism that are not restricted to their co-binding at enhancers, suppressors, and promoters. They also play architectural roles, activate chromatin remodeling and block nucleosome repositioning^93^. However, there is still a limited understanding of the link between transcription factor enrichment and stochasticity in gene expression. Our results showing the contribution of various transcription factors on gene expression distribution provide important data to this fundamental question. Interesting, we found several pioneer transcription factors underlie expression noise observed in our study. A better understanding of genetic characteristics and genomic domains that favor the binding of those pioneer factors will shed light on detailed mechanisms of how chromatins connect with transcription factors to modulate gene expression.

We noted that transcription factors found to influence reporter expression mean and noise in our study might be specific to the CMV promoter. Future work that generalizes our findings to other promoters is an important next step. The methodological advantages in the creation of scalable assays we describe will be key to addressing these issues. Another key limitation of our results is that they are only based on correlation without the direct establishment of causation. It is possible that fluctuations in gene expression cause specific recruitment of histone^94^. Specific manipulation of chromatin state followed by a functional assessment of change in reporter gene expression will be needed to address this question. Nonetheless, given the complexity of chromatin states and the technical challenges associated with their manipulation^95^ the initial step of the establishment of correlation is a vital first step in this path.

The establishment of a scalable assay to measure chromatin positional effects on gene expression distribution is a key stepping stone to understanding chromatin regulation of gene expression variability. The presented results generate many intriguing hypotheses related to chromatin regulation of gene expression distributions. Given the recent interest in the development of pharmacological approaches to manipulate gene expression variability and the ongoing efforts to find more drug leads^62^, the mechanistic leads identified here can point towards the mechanism of action of some of these drugs.

## Methods

### Experimental model and subject details

#### Cell lines

K562 cells were maintained in RPMI media (Gibco) supplemented with 10% FBS (Gibco), 1% GlutaMAX(Gibco) and 1% Penicillin Streptomycin. Cells were grown at 37 °C in an incubator with 5% CO_2_. HEK 293 T cells for viral packing were grown under the same conditions in DMEM (Gibco) supplemented with 10% FBS (Gibco), 1% GlutaMAX(Gibco) and 1% Penicillin Streptomycin.

### Method details

#### Library construction of barcoded reporter plasmid

The library was constructed as described previously^98^. The master plasmid excluding barcode was first constructed to contain the following essential elements. Lentiviral production units include HIV-1 truncated 5′ LTR, HIV-1 packaging signal, HIV-1 Rev response element (RRE), HIV-1 truncated 3′ LTR and Central polypurine tract (cPPT). These components allow proper viral packaging and viral integration into host cells. As a transcription unit, we used cytomegalovirus promoter (CMV) to drive expression of the reporter gene encoding yellow-green fluorescent protein (mClover). Woodchuck hepatitis virus posttranscriptional regulatory element (WPRE) is placed after mClover to enhances mRNA stability and protein yield. Ampicillin resistance gene (β-lactamase) is included for selection of plasmid in bacterial cells.

To generate barcoded plasmid libraries, based lentiviral plasmid was cut upstream of the CMV promoter by ClaI restriction enzyme and purified by ethanol precipitation. The inserted cassette of 127-bp-long oligonucleotide containing a random 16-bp-long barcode sequence (repeats of A,T and G), MspI site, primer priming site and homology arms, were synthesized by Integrated DNA Technology. The assembly reaction of 1:5 vector:insert ratio was carried out for 1 h at 50 °C using NEBuilder HIFI DNA assembly kit (New England Biolabs, NEB). Assembly products were electroporated into NEB Turbo Competent E.Coli (NEB) and then plated on ampicillin-containing medium. Ampicillin resistant colonies were collected and extracted for plasmids using Maxiprep kit (Invitrogen). Ten sampling clones from the agar plate were analyzed by PCR with forward primer, GATCCTGTAGAACTCTGAACCT, and reverse primer, AGTCGGTGTCTTCTATGGAG, and Sanger sequencing to verify successful cassette insertion and barcode diversity.

#### Generation of reporter cell lines

The lentiviral library carrying the barcoded reporter cassettes was used to transduce into K562 cells at a multiplicity of infection of 0.01 by culturing cells with barcoded virus in media supplemented with 5 μg/ml polybrene and 20 mM HEPES for 2 h of spinoculation and 24 h of incubation. Afterwards, cells were collected by gentle centrifugation and the media was replaced with fresh cultured media. Cells were expanded for 72 h and then subjected to FACS to isolate Clover-positive founder cells. Founder cells were expanded for 14 days and split into two pools. One was used for genomic mapping of barcoded reporter and the other was used for establishing isogenic reporter clones by single cell sorting.

#### Measurement of reporter protein expression

A million cells were passed through a 35 μm mesh filter (Corning 352,235) and placed on ice prior to FACS separation. Cells are resuspended in FACS buffer consisting of 96% PBS, 2% fetal bovine serum, 1% 100X Pen/Strep, and 1% 0.5 M EDTA (pH 7.4). 50 k live cells were collected from each well for FSC-A, SSC-A, the Clover intensity and IRFP intensity using the BD FACSCelesta flow cytometer. Data were analyzed with custom Matlab scripts. A very conservative gating for a live subset of ~ 3 k cells of similar size, volume, and state, was applied on the FSC versus SSC to reduce extrinsic noise contributions.

#### Identification of genuine barcodes and genomic integration sites

Identification of genuine barcodes was performed as previously described^98^. A library of genuine barcodes in founder cells was first listed. Briefly, barcode region was amplified in first nested PCR from 5 µg of genomic DNA in 50 ul of 20 cycle PCR reaction using Titanium Taq, forward primer: TATGGATCCTGTAGAACTCTG, and reverse primer: GCTCTGCTTATATAGACCTCCCAC. Barcode amplicons were enriched from genomic DNA using SPRI beads(Beckman Coulter) and further amplified in the second nested PCR for 20 cycles using forward primer: TGTAGAACTCTGAACCTAGCT and reverse primer: CGTAAGTTATGTAACGCGGA. Illumina adapter was attached to final amplicon, amplified and sequenced on Illumina HiSeq 3000 platform (1 × 50 bp). Sequencing reads were filtered and analyzed using Matlab Bioinformatics Toolbox. To identify genuine barcode, we used the following algorithm. First, we sorted barcodes according to their counts from most frequent to least frequent. Then, mutant versions of each barcode, defined as barcodes within a Hamming distance of 2, were sequentially removed. We consider remaining sequences as ‘‘genuine’’ barcodes. We recovered 756 genuine barcodes from 3,000 sorted founder cells.

Mapping of reporter integration sites was done by inverse PCR coupled with high-throughput sequencing. Briefly, founder cells were collected and splitted into two replicates. For each replica, 2 µg of genomic DNA was digested with 20 units of MspI (NEB) overnight at 37 °C in a volume of 100 µl. Subsequently, three sets of ligation reactions were set up by incubating 600 ng of purified digested DNA with 2 µl of high-concentration T4 DNA ligase (NEB, M0202T) overnight at 4 °C in a volume of 400 µl. The ligation reactions were purified by phenol–chloroform isoamyl alcohol extraction and ethanol precipitation. DNA pellets were dissolved in 30 µl of water. Two rounds of PCR were performed to amplify and enrich fragments containing both the barcodes and flanking genomic DNA regions. For the first round of nested PCR, five sets of 25-cycle reaction in a volume of 50 µl were performed using 5 µl of ligated products as templates, forward primer: TATGGATCCTGTAGAACTCTG, reverse primer: GCTTCAGCAAGCCGAGTCCTGCGTCGAG and Phusion Hot Start Flex 2X Master Mix (NEB). Amplicon was pooled together, cleaned by DNA Clean & Concentrator kit (Zymo), and diluted in 50 ul of water. For the second round of nested PCR, four sets of 15-cycle reaction in a volume of 50 ul were done with 5 ul of cleaned amplicon from first PCR, forward primer:TGTAGAACTCTGAACCTAGCT, reverse primer:GCTTTCAGGTCCCTGTTCGG. Purified sample was further ligated with Illumina adapter, amplified and sequenced on Illumina HiSeq 3000 platform (2 × 150 bp). Sequencing reads were filtered and analyzed using Matlab Bioinformatics Toolbox. The genomic regions associated with genuine barcodes were extracted from mapping reads and aligned against the human genome (hg38) using STAR^96^. Detected integration sites from each replicate were compared and assigned to each genuine barcode only if top candidate site from both replicates are identical. Genome coordinate of reporter integration site was converted to human reference genome(hg19) using UCSC liftOver tool^97^ for comparison to ChIP-Seq data.

#### Combinatorial pool sequencing

Combinatorial pooled sequencing was performed as previously described (Zhang 2020). To simultaneously reveal the identity of reporter cell lines linked by DNA barcodes in a single run, combinatorial pooled sequencing was used. Specifically, clonal numbers were encoded in a form of pooling pattern and DNA barcodes were decoded from such known pattern. To increase decoding accuracy, we designed pooling signature to be unique four selected pools out of total eighteen pools. Cells from each clone were split into four selected pools according to the design. Sequentially, genomic DNA from individual pool of mixed clones was extracted and used as templates for PCR to amplify barcode using same procedure described in the method of identification of genuine barcode list. Forward primers of second nested PCR contain 6-bp index DNA to label PCR products from each pool, which allow high-throughput multiplex sequencing. Sequences were filtered and demultiplexed using Matlab Bioinformatics Toolbox. Genuine barcodes from all pools were first listed. For each detected barcode, normalized counts per pools were calculated and pools showing high reads above the threshold were identified. Barcodes with four detected pools were first assigned to the clone showing matched pooling design. Some barcodes were found in more than four pools when sister cells, expanded from one founder cell, were sorted into multiple wells during single-cell sort. A list of merged pooling signature of two unassigned clones was matched with barcodes showing complexed readout. Clones with two inserted barcodes(~ 2% of the population) were excluded from the library of reporter cell lines.

#### Validation of Combinatorial pool sequencing

For the validation of combinatorial pool sequencing, 5 clones were randomly chosen and extracted for genomic DNA using PureLink Genomic DNA Mini Kit (Invitrogen). 200 ng of purified genomic DNA was used as a template for PCR amplification with a set of validation primers, forward:TAGTGAACGGATCTCGACG, reverse:GCTCTGCTTATATAGACCTCCCAC. PCR products were cleaned by DNA Clean & Concentrator kit (Zymo) and Sanger sequenced to verify the barcode sequences.

### Quantification and Statistical Analysis

#### Sequencing, quality control and demultiplexing

Libraries were sequenced on Illumina Hiseq3000 sequencing systems by UCLA technology center for genomics & bioinformatics. Low quality sequences were filtered out by mismatched read length and low quality scores(> 25% of base with score < 20) using Matlab Seqfilter. Sample indexes were trimmed with Matlab Seqtrim and reads from different samples were demultiplexed by Matlab Seqsplit.

#### Multivariate linear regression analysis

Fluorescence data of each reporter cell line was collected in triplicate from different experimental setup. The mean and CV^2^ of Clover intensity was calculated from a population of more than 3,000 cells with stringent control of size, volume and state for each replica. We used the average values of mean and CV^2^ across the three replicas for downstream analysis. We used the average values across the three replicas due to the high degree of agreement between them. The correlations values for the mean expression between replicas 1–2,1–3,2–3 were 0.98, 0.96, 0.96. A similar agreement was seen for CV^2^ where the pairwise values were 0.82, 0.77, 0.8. To understand the relationship between TF enrichment and reporter expression, Spearman's rank-order correlation coefficient between Clover mean or CV^2^ and averaged transcription factor enrichment within a window of 50 kb was first assessed. The size of 50 KB was chosen as a tradeoff between the size of TADs (~ 100KBs, Akhar 2013) and the need for increased signal. When the window size is too small (5 KB) preliminary analysis showed a very high degree of variability motivating a larger window size. However, a window size that is comparable to the size of a TAD will not reflect the local environment anymore and therefore we chose the size of 50 KB. We used K562 ChIP-Seq datasets of transcription factor in the format of fold change over control, uniformly processed from two replicates, from the ENCODE database (Michael Snyder,Stanford) at https://www.encodeproject.org. To determine which features (transcription factors or chromatin states) are meaningful we used a standard multivariate linear regression. Out of possible 200 transcription factors, we only included the subset in the model with an indication that they might be informative. The criterion we chose was Spearman correlation > 0.2. This allowed 19 features in the model that tested for changes in mean expression and 37 independent variables in the model that tested for CV2. The analysis of statistical significance was done using standard multivariate regression analysis (using the Matlab command fitlm). Chromatin state data was obtained from Genome Segmentations from ENCODE (ChromHMM Segmentations) at UCSC (Data version: ENCODE Jan 2011 Freeze).

## Data and materials availability

Further information and requests for resources and reagents should be directed to, and will be fulfilled by the corresponding author Roy Wollman (rwollman@ucla.edu).
